# 

**Published:** 1879-11

**Authors:** 


					﻿Whole Number,
CCXX.
NOVEMBER, 1879.
Number 4,
Volume XIX.
THE
BUFFALO
Medical and Suraical Journal.
EDITORS:
THOS. LOTHROP, M.
r. W. VAN PEYMA, M.
A. R. DAVIDSON, M.'D.,
HERMAN MYNTER, M.
LVCIEN HOWE, M. D.
BUFFALO :
Published by the Medical Journal Association.
Read the Notice of this Journal among the Advertisements.
Entered at the Post-Office at Buffalo, N. Y., as Second-Class Mail Matter.
				

